# Prefrontal cortex activation during story encoding/retrieval: a multi-channel functional near-infrared spectroscopy study

**DOI:** 10.3389/fnhum.2013.00925

**Published:** 2013-12-31

**Authors:** Sara Basso Moro, Simone Cutini, Maria Laura Ursini, Marco Ferrari, Valentina Quaresima

**Affiliations:** ^1^Department of Life, Health and Environmental Sciences, University of L’AquilaL’Aquila, Italy; ^2^Department of General Psychology, University of PaduaPadova, Italy

**Keywords:** verbal working memory, encoding, retrieval, cortical oxygenation, functional near-infrared spectroscopy, prefrontal cortex

## Abstract

Encoding, storage and retrieval constitute three fundamental stages in information processing and memory. They allow for the creation of new memory traces, the maintenance and the consolidation of these traces over time, and the access and recover of the stored information from short or long-term memory. Functional near-infrared spectroscopy (fNIRS) is a non-invasive neuroimaging technique that measures concentration changes of oxygenated-hemoglobin (O_2_Hb) and deoxygenated-hemoglobin (HHb) in cortical microcirculation blood vessels by means of the characteristic absorption spectra of hemoglobin in the near-infrared range. In the present study, we monitored, using a 16-channel fNIRS system, the hemodynamic response during the encoding and retrieval processes (EP and RP, respectively) over the prefrontal cortex (PFC) of 13 healthy subjects (27.2 ± 2.6 years) while were performing the “Logical Memory Test” (LMT) of the Wechsler Memory Scale. A LMT-related PFC activation was expected; specifically, it was hypothesized a neural dissociation between EP and RP. The results showed a heterogeneous O_2_Hb/HHb response over the mapped area during the EP and the RP, with a O_2_Hb progressive and prominent increment in ventrolateral PFC (VLPFC) since the beginning of the EP. During the RP a broader activation, including the VLPFC, the dorsolateral PFC and the frontopolar cortex, was observed. This could be explained by the different contributions of the PFC regions in the EP and the RP. Considering the fNIRS applicability for the hemodynamic monitoring during the LMT performance, this study has demonstrated that fNIRS could be utilized as a valuable clinical diagnostic tool, and that it has the potential to be adopted in patients with cognitive disorders or slight working memory deficits.

## Introduction

Encoding, storage and retrieval constitute three fundamental stages in information processing and memory. Encoding is the initial elaboration of input data, that allows the creation of new memory traces; storage refers to the maintenance and the consolidation of new memory traces over time, while retrieval refers to the process of accessing and recovering stored information from short or long-term memory (Buckner and Koutstaal, 1998; for a review see Cabeza and Nyberg, 2000). Short-term memory allows the temporary storage of a limited quantity of information previously encoded (Atkinson and Shiffrin, 1968), and the processes of active maintenance and rehearsal allow to not forget the information and to hold them for a short period of time (from few seconds to hours). A wide range of complex cognitive activities as reasoning, language comprehension, planning, and spatial elaboration, requires the combination of the short-term storage and the manipulation of the information, processes that, taken together, are defined as working memory (WM; Baddeley, 2012). Indeed, WM is a core component of human cognition, being an essential part of the mnemonic processes, and fundamental for many cognitive activities (Baddeley and Hitch, 1974; Petrides, 1989; Fletcher and Henson, 2001). Among the several theories regarding the cognitive structure and functioning of WM, the Baddeley’s model (Baddeley and Hitch, 1974) is likely to be the most influential one. Such model holds that WM is based on a supervisory system, the central executive, and three subsystems, each one specialized in the maintenance and manipulation of different types of information. The phonological loop deals with verbal information; the visuo-spatial sketchpad with spatial and visual information; the episodic buffer with episodic information (for a review see Baddeley, 2012). Other major theories of WM (e.g., Kane et al., 2001; Cowan, 2005; Oberauer, 2009) underline the importance of the attentional role in controlling the activation, maintenance, and manipulation of short-term internal representations. So, rather than a short-term store, WM is considered as a limited-capacity attentional system, that interacts both with perception and with long-term memory, allowing the construction of new representations (Oberauer, 2009). Indeed, attention and WM are increasingly viewed as overlapping constructs (Kane et al., 2001; Postle, 2006; Gazzaley and Nobre, 2012). Functional imaging has provided considerable evidence about the neural correlates of WM processes, showing that they reside in prefrontal cortex (PFC) (for review see D’Esposito et al., 2000; Fletcher and Henson, 2001). In particular, ventrolateral prefrontal cortex (VLPFC) is more often activated during tasks requiring maintenance (left VLPFC seems to be engaged in the maintenance of verbal information, and right VLPFC in the maintenance of spatial information), while dorsolateral prefrontal cortex (DLPFC) is more often activated during tasks requiring manipulation (for a review see Fletcher and Henson, 2001). This is consistent with Petrides’ model (Petrides, 1989), which states that VLPFC initially receives and organizes the information, whereas DLPFC is additionally recruited only when monitoring and manipulation of information within WM is required. Imaging studies have also supported the dissociation between storage and rehearsal. The DLPFC and the anterior frontal regions would be associated with executive control of WM, as well as manipulation processes on the information already maintained in memory; the anterior frontal regions seem to be associated with maintaining the goals and products of one task while performing another (Fletcher and Henson, 2001). From a neural perspective, encoding and retrieval processes share some cortical structures (Rugg et al., 2008), even though it is still debated to what extent their neural circuitries overlap, and how much they depend on their own specific features, such as the type of encoded and retrieved material (Kelley et al., 1998). For instance, the encoding-related cortical activity seems to reflect the cognitive load elicited by the task, thus depending on the nature of the online processing demands (Otten and Rugg, 2001; Uncapher et al., 2006). Moreover, it seems that when the neural activity elicited during retrieval engages the same processing involved during encoding, a more successful retrieval is performed (Craik, 2002; Rugg et al., 2008). Encoding and retrieval can be experimentally investigated by presenting subjects different kinds of stimuli (such as words, pictures or narratives), that have to be learned and subsequently recalled (Cabeza and Nyberg, 2000). While words and pictures have been extensively used in memory research, much less studies have employed narratives. A narrative presentation is the description of a sequence of actions or events that follow one another over time, on the basis of causal principles (Graesser et al., 1980). Both narrative comprehension and production involve a number of identical cortical areas, including medial and dorsolateral regions of the frontal cortex. Moreover, the activation pattern observed in narrative processing seems to be different from the activation pattern elicited in word recognition and production or sentence-level operations (Crozier et al., 1999; Cabeza and Nyberg, 2000; Robertson et al., 2000). In narrative comprehension, as reviewed by Mar (2004), some regions appear to be critical: the frontopolar cortex (FPC), that supports the maintenance of the information; the DLPFC, associated with temporal ordering and integration, processing of sequential information, and monitoring/manipulating the contents of WM; the VLPFC, that seems to play a role in specification and/or maintenance of cues for long-term retrieval and encoding. Finally, it should also be noted that frontal areas modulate the attentional system by enhancing the recruitment of other specific cortical areas (Fuster, 2002). Pertaining to narrative production, neuroimaging studies showed a smaller body of evidence. Activations have been found in medial and dorsolateral frontal gyri, temporoparietal junction, and the posterior cingulate (Braun et al., 2001). Importantly, one critical aspect in narrative comprehension and production tasks is the normal functioning of encoding and retrieval processes, because they enable to encode incoming stimuli, rehearse received input, assess and retrieves stored knowledge, thus allowing all WM operations (Bayles, 2003). Recent researches have examined the potential clinical use of oral narratives to identify language impairments, in pediatric psychiatric population (Pearce et al., 2013) or in people suffering of mild cognitive impairments (Shankle et al., 2005; Roark et al., 2011). Being able to validly assess communicative and linguistic abilities through standardized tests is important for the clinical diagnostic evaluation of patients with cognitive disorders, memory impairments or slight WM deficits. Moreover, the possibility to monitor the neural activation elicited by the task provides an important contribution in cognitive functioning comprehension.

In the present study, we used the Logical Memory Test (LMT) of the Wechsler Memory Scale (Wechsler, 1997) as a spoken language derived measure to investigate the encoding and retrieval processes (EP and RP, respectively). So far, the LMT has been widely used as a clinical assessment measure of WM, because it represents an index of auditory-linguistic memory, requiring the immediate and delayed verbal recall of auditorally presented three-sentence verbal narrative containing 24 mnemonic units (Abikoff et al., 1987). Here, we recorded the hemodynamic activity of PFC using functional near-infrared spectroscopy (fNIRS), a functional brain imaging technique that, similarly to functional magnetic resonance imaging (fMRI), monitors hemodynamic changes in the cerebral cortex (see Cutini et al., 2012a; Ferrari and Quaresima, 2012, for reviews). However, unlike the blood-oxygen-level-dependent (BOLD) signal of fMRI, which is gathered from the paramagnetic properties of deoxygenated-hemoglobin (HHb), fNIRS is based on the intrinsic optical absorption of blood. As a result, fNIRS can simultaneously record the variations of HHb and oxygenated hemoglobin (O_2_Hb) concentrations. We investigated, with a 16-channel fNIRS system, the temporal and spatial characteristics of the hemodynamic PFC activity in healthy subjects performing the LMT. We expected a LMT-related PFC activation, given the major involvement of the PFC in WM processing. Specifically, we hypothesized a neural dissociation between EP and RP, given: (i) the contributions of the VLPFC in the semantic maintenance of verbal information, in the narrative comprehension, and in the maintaining memory cues for long-term encoding, and (ii) the DLPFC role in monitoring and manipulating the memory traces, in processing the sequential information giving them a temporal order and integration.

## Materials and methods

### Participants

Thirteen healthy subjects (27.2 ± 2.6 years; high level of education) participated in the study. Only men were recruited to avoid possible gender differences in emotional responses. Informed consent was obtained after a full explanation of the protocol and the non-invasiveness of the study. To exclude left-handed subjects, all participants completed the Edinburgh Handedness Inventory (Oldfield, 1971) assessing hand dominance.

### Experimental setup

#### Functional near-infrared spectroscopy (fNIRS) instrumentation and signal processing

A 16-channel continuous wave fNIRS system (Oxymon Mk III, Artinis Medical Systems, Netherlands) was employed to map changes in O_2_Hb and HHb over bilateral PFC. This device measures changes in light attenuation at two wavelengths, 764 and 858 nm, and utilizes the modified Beer-Lambert law with an age-dependent differential pathlength factor to resolve changes in O_2_Hb and HHb concentrations within cortical brain tissue. Six optical fiber bundles (length: 3.15 m; diameter: 4.5 mm) were utilized to carry out the light to the left and the right PFC (three for each hemisphere), whereas eight optical fiber bundles of the same size (four for each hemisphere) were utilized to collect the light emerging from the same cortical areas. The illuminating and collecting bundles were assembled into a specifically designed flexible probe holder ensuring that the position of the 14 optodes, relative to each other, was firmly fixed. The probe holder consisted of two mirror-like units (9.7 × 8.9 cm each) held together, along the longest side, by three flexible junctions. The detector–illuminator distance was set at 3.5 cm. The optodes were inserted into apolyoxymethylene probe holder by connectors. The probe holder was appropriately placed over the head in order to include the underlying PFC. In particular, the two frontopolar fibers bundles collecting light at the bottom of the holder were centered (according to the International 10–20 systems for the EEG electrode placement) at the Fp1 and Fp2 for right and left side, respectively. The MNI (Montreal Neurological Institute) coordinates of the optodes and the relative 16 measurement points (channels) were calculated using a probe placement method (Cutini et al., 2011) based on a physical model of the head surface of ICBM152 (Mazziotta et al., 2001) (the standard brain template in neuroimaging studies) and a 3D digitizing system (BrainSight™, Rogue Research). As a final step, the MNI coordinates of each channel on the right hemisphere have been averaged with their symmetrical ones in the left hemisphere (1–9, 2–10, 3–11, 4–12, 5–13, 6–14, 7–15, 8–16). This procedure has allowed us to provide a broad estimate of the average error made during the probe placement. Notably, the mean difference in absolute values between left and right channels was 3.4 mm, well below the spatial resolution achievable with the present setting. Afterwards, in order to identify the corresponding Brodmann Areas (BAs), the measurement points were overlaid onto the Brodmann template using MRIcron software.^1^ The investigated BAs were then: BA 9 (measurement points: 1, 3, 9, 11), BA 46 (measurement points: 2, 4, 5, 10, 12, 13), BA 10 (measurement points: 6, 8, 14, 16), and BA 45 (measurement points: 7, 15).

The probe holder was fixed over the head by a velcro brand fastener, adapting it to the individual size and shape of the different heads. This flexible probe holder and its position on the head provided a stable optical contact with the scalp for all optodes. The accuracy of the contact between the optodes and the scalp was verified at the end of the protocol. The pressure created by the velcro brand fastener was adequate to induce a partial transient blockage of the skin circulation during the fNIRS study, as witnessed by the presence of the well-defined 14 circles over the PFC skin (depressed cutaneous areas associated with the location of the 14 optodes).

The 14 circles over the forehead skin started to disappear 15–20 min after the end of the protocol. The adopted procedure would suggest that a consistent reduction of forehead skin blood flow was occurring as a result of this approach (Takahashi et al., 2011).

The O_2_Hb and HHb data from the sixteen measurement points, which are defined as the midpoint of the corresponding detector–illuminator pairs, were acquired at 10 Hz. During the data collection procedure, O_2_Hb and HHb concentration changes were displayed in real time, and the signal quality and the absence of movement artifacts were verified. The coming out concentration changes in O_2_Hb and HHb, calculated according to a modified Beer-Lambert Law, were transferred online from the fNIRS system to a personal computer (OxySoft DAQ 2.1.6, Artinis Medical Systems, Netherlands). The additional quantification of the concentration changes (expressed in ΔμM) of O_2_Hb and HHb was obtained by including an age-dependent constant differential pathlength factor (4.99 + 0.067*age^0.814^) (Duncan et al., 1996). In order to remove the drift introduced either by the system or by any possible spontaneous baseline fluctuations over the protocol (Scholkmann et al., 2014), the time series data of O_2_Hb and HHb concentration changes were first detrended utilizing the function “detrend” of the commercial numerical and statistical package MATLAB (MathWorks, Natick, MA).

A 2-s moving-average filter was applied to attenuate cardiac signal, respiration, and Mayer-wave systemic oscillations. The subject’s heart rate (HR) was monitored by a pulse oximeter (N-600, Nellcor, Puritan Bennett, St. Louis, MO) with the sensor clipped to the index finger of the left hand.

#### Experimental design

**Figure 1:**
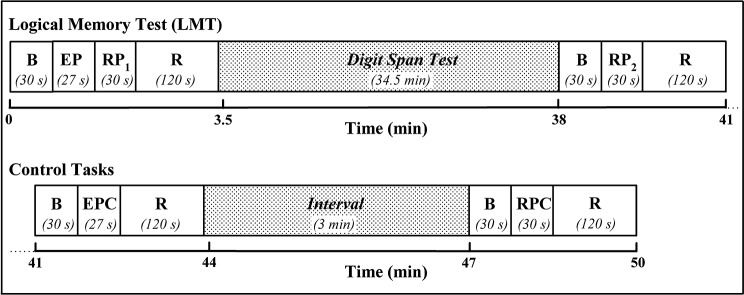
**Scheme depicting the experimental protocol**. B: baseline; EP: encoding process (subjects’ verbal presentation of the LMT-story); RP: retrieval process (subjects’ aloud recall of the story elements); R: recovery; EPC: encoding process control (subjects’ verbal presentation of the backward LMT-story); RPC: retrieval process control (subjects’ week day aloud sentence repetition).

Prior to the study, subjects were informed about the procedures and familiarized with the protocol. Subjects were asked to sit on a comfortable chair in front of a 17″ PC monitor placed at a distance of 70 cm. The schematic illustration of the experimental protocol is reported in Figure 1. Specifically, the protocol started with a 30-s baseline period, in which participants were asked to relax, in order to get stable baseline fNIRS signals. Then, visual instructions informed the participants that the 27-s story of the LMT (Wechsler, 1997) would be acoustically presented (EP). Immediately after the end of the recording, a beep-tone and a visual instruction alerted the subjects to repeat the story aloud trying to recall as many details as possible (RP). The RP lasted 30-s after which participants were acoustically instructed to relax until the end of the 2-min recovery period. About 35 min later, a second RP, preceded by a beep-tone and a visual instruction, occurred. Once again the subjects were asked to repeat the story aloud trying to recall as many details as possible. During the 34.5-min period, subjects were distracted by performing a visual modified version of the digit span test (Wechsler, 2008). Two-min after the end of the second RP, two control tasks were administered to the subjects: (1) a 27-s backward story for the EP (EP control, encoding process control (EPC)), and (2) a 30-s week day aloud sentence repetition for the RP (RP control, retrieval process control (RPC)). The backward story consisted in presenting the previous story transformed backward (Audacity^®^ 2.0, the Free, Cross-Platform Sound Editor), while the sentence repetition consisted in repeating self paced and aloud the week days. The baseline and the recovery periods lasted 30 and 120 s, respectively; the interval between EPC and RPC was set at 3 min. The verbal responses were recorded for each subject who vocalized as softly as possible to reduce movement related artifacts but loud enough to convey the response to the experimenter and the recorder. A commercially available software package (SuperLab Pro Edition 4.5 Executable, Cedrus Corporation, Canada) was used to present visual and auditory stimuli and instructions.

At the end of the protocol, subjects completed the DP-15 rating scale for the perceived difficulty (Delignières, 1993), attributing to the LMT a value from 1 to 15 (where 1 corresponds to “very, very easy”; 6 to “easy”; 10 to “difficult”; 15 to “very, very difficult”). In order to evaluate the “state anxiety”, the subjects completed 20-items of the State-Trait Anxiety Inventory (STAI)-Form Y-1 before and after the protocol (Spielberger et al., 1983).

### Data analysis and statistics

#### Performance evaluation and heart rate (HR) data analysis

The LMT score (performance) was obtained matching the results of the two RP, calculating the mean number of the two summary scores (the raw number of elements recalled) (Wechsler, 1997). The mean values of the HR changes during the study were calculated every 5 s. A one-way repeated measures analysis of variance (ANOVA) was performed in order to evaluate the influence of the time on HR changes.

#### Functional near-infrared spectroscopy (fNIRS) data analysis

The maximum values of the concentration changes in O_2_Hb and HHb over the PFC were obtained from the mean values, calculated every 5 s, of the EP/RP data after subtracting the respective control tasks (EPC/RPC). These resulting maximum values were baseline-corrected. The baseline was calculated as follows: (1) the mean value over the last 10 s of the baseline period for the EP and for the EPC/RPC; and (2) the mean value over the last 10 s of the EP for the RP. In order to investigate the PFC activation in response to the EP and the RP, a repeated measures ANOVA was performed for O_2_Hb/HHb maximum values. The ANOVA analysis included two factors: channel (8 levels) and hemisphere (2 levels). A one-way repeated measures ANOVA was performed to evaluate the influence of the channel on O_2_Hb/HHb maximum values during the EP/RP. Student’s *t*-tests were conducted in order to evaluate the presence of any difference over the 16 measurement points between the O_2_Hb/HHb maximum values of the EP vs. the EPC and the RP vs. the RPC. In order to check for the presence of detectable PFC activation during the control tasks (EPC/RPC), a repeated measures ANOVA was performed for O_2_Hb/HHb maximum values. The ANOVA analysis included two factors: channel (8 levels) and hemisphere (2 levels).

The Pearson’s correlation coefficient was calculated to evaluate the relation between: (1) individual performance and O_2_Hb/HHb changes (mean of the 16 measurement point maximum values) during the RP; and (2) the subjects’ HR (mean of the EP/RP maximum values) and O_2_Hb/HHb changes (mean of the 16 measurement point maximum values) during the EP/RP.

All statistical analyses were conducted with SPSS 20.0 (SPSS Inc., Chicago, IL). Data were expressed as mean ± SD. The criterion for significance was *p* < 0.05.

## Results

**Figure 2:**
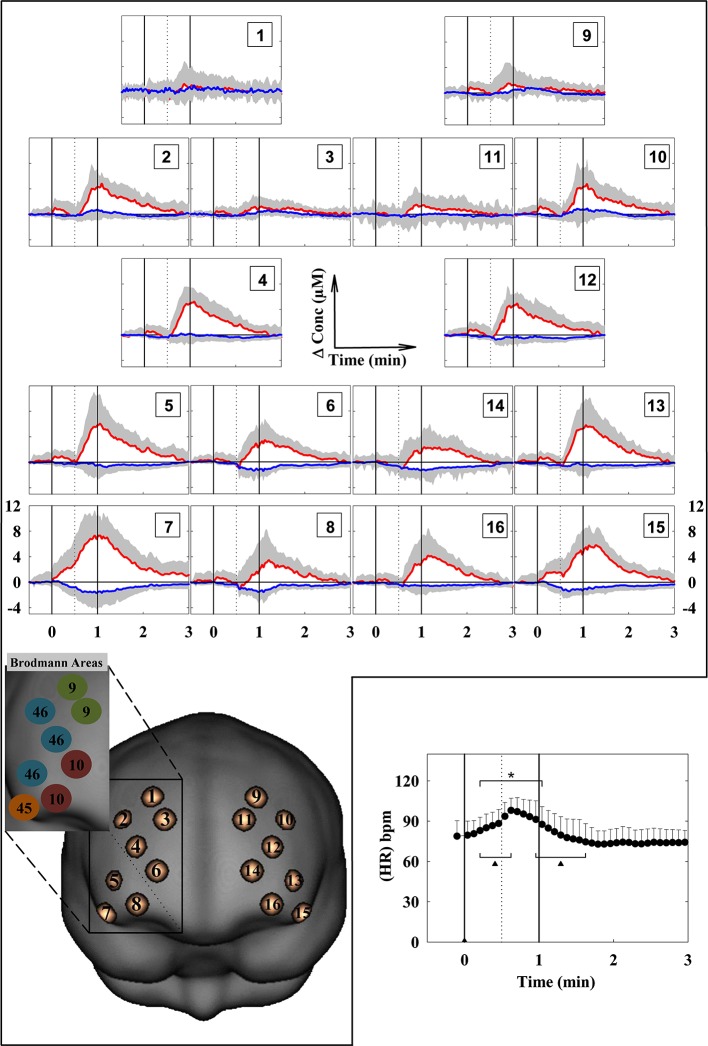
**Grand average of the cortical oxygenation changes (increase in O_2_Hb and decrease in HHb) over the prefrontal cortex and heart rate (HR) changes (lower right panel) during the LMT**. The numbers 1–16 of the panels refer to the cerebral projections of the measurement points superimposed on the ICBM152 template brain. The points have been created with a 1-cm Gaussian blurring, to reproduce the spatial resolution of fNIRS. The vertical solid lines limit the LMT. The dotted vertical line limits the EP and RP. HR changes were calculated every 5 s. bpm: beats per minute. (*n* = 13; mean ± SD). * *p* < 0.05 with respect to the baseline. ^▲^ *p* < 0.05 with respect to the previous value.

The behavioral data analysis revealed the following main results. The recalled elements of the LMT were 25 ± 10, falling within the equivalent range of 4, considered as a normal performance. The mean subjective rating of perceived difficulty during the LMT was 6.9 ± 2.5, suggesting that the task was considered by the participants as “somewhat difficult”. The difference in the “state anxiety” before (31.0 ± 6.8) and after the protocol (28.0 ± 4.8) was not significant (*t* = 1.304, *p* = 0.205). HR started to increase 15 s after the beginning of the EP, reaching its maximum value (around 125% of baseline) 10 s after the beginning of the RP. Then, HR progressively declined, returning to the baseline value 5 s after the end of the RP (Figure 2). The ANOVA carried out on HR changes, revealed a significant main effect of time (*F*_(36,432)_ = 17.188, *p* < 0.001).

**Figure 3:**
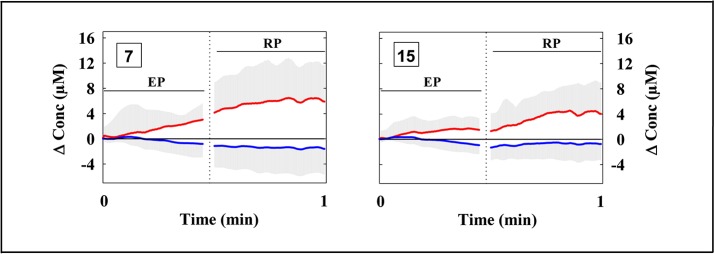
**Grand average of the cortical oxygenation changes over the ventrolateral prefrontal cortex (channels 7 and 15) during the LMT after the subtraction of O_2_Hb and HHb changes occurring during the corresponding control tasks (see Section Materials and Methods)**. The two vertical dotted lines limit the end of the EP and the beginning of the RP. (*n* = 13; mean ± SD).

The fNIRS data evidenced a heterogeneous O_2_Hb/HHb response over the mapped area during both the EP and the RP (Figure 2). O_2_Hb progressively increased since the beginning of the EP in most of the measurement points, then progressively decreased in the second part of the EP; this O_2_Hb increase was more evident in the measurement points 7 and 15, corresponding to the VLPFC (Figure 3). Since the beginning of the RP, O_2_Hb increased consistently and progressively in most of the measurement points up to 10–15 s after the end of the RP. Then, O_2_Hb progressively decreased returning to the corresponding baseline values within the end of the considered recovery time. During the LMT, HHb changes were smaller than O_2_Hb changes.

The ANOVA analysis, carried out on O_2_Hb changes during the EP, revealed a significant main effect of the channel (*F*_(3.16,37.87)_ = 3.177, *p* = 0.033). The ANOVA analysis carried out on HHb changes during the EP revealed no significant main effects nor interaction (all *ps* > 0.05). The series of channel-wise *t*-tests for O_2_Hb revealed a significant difference in channels 1, 7, and 15 in the comparison between EP and EPC. In particular, the channels 7 and 15 showed the highest *t*-value (*t* = 2.742, *p* = 0.018; *t* = 2.717, *p* = 0.019). The *t*-tests were limited to O_2_Hb because of the lack of significant effects in the ANOVA for HHb.

The ANOVA analysis, carried out on O_2_Hb changes during the RP, revealed a significant main effect of the channel (*F*_(7,84)_ = 4.82, *p* < 0.001). The ANOVA analysis, carried out on HHb changes during the RP, revealed a significant main effect of the channel (*F*_(3.43,41.20)_ = 2.858, *p* = 0.042). The series of channel-wise *t*-tests for O_2_Hb revealed a significant difference in channels 2, 3, 4, 5, 7, 8, 10, 12, 13, 14, 15, and 16 (*ps* < 0.05) in the comparison between RP and RPC. For HHb, the channel-wise *t*-tests revealed a significant difference in channel 9 only (*t* = 2.595, *p* = 0.023).

The ANOVA analysis carried out on O_2_Hb/HHb changes during the control tasks (EPC/RPC) revealed no significant main effects nor interaction (*ps* > 0.05).

No correlation was found between subjects’ performance and the RP PFC changes in O_2_Hb/HHb (*r* = 0.543, *p* = 0.055; *r* = −0.249, *p* = 0.412, respectively). No correlation was found between subjects’ HR and the corresponding EP/RP PFC changes in O_2_Hb (*r* = 0.336, *p* = 0.262; *r* = −0.403, *p* = 0.172, respectively) and in HHb (*r* = 0.045, *p* = 0.885; *r* = 0.312, *p* = 0.300, respectively).

## Discussion

To the best of our knowledge, this is the first time in which the LMT has been utilized in a fNIRS neuroimaging study. The hemodynamic results evidenced a moderate, but focused, activation in the bilateral VLPFC (measurement points 7 and 15) during the EP (Figure 3), and a broader activation in the bilateral VLPFC, DLPFC and FPC during the RP (Figures 2, 3). These findings support our hypotheses and are consistent with the previous neuroimaging studies in which PFC is considered as the neural substrate of WM (D’Esposito et al., 2000; Fletcher and Henson, 2001). In particular, the prominent activation found in channels 7 and 15, corresponding to the right and left VLPFC, is in line with its role in the selection, comparison, or decision about the information held in memory (Petrides, 1989), and in the specification and/or maintenance of cues for long-term encoding (Mar, 2004). The VLPFC recruitment could be explained by the evidence that many cognitive processes are required in speech comprehension: both the semantic representations induced by the narrative stimulus, and the strategic/executive/control processes that are required to access, retrieve, compare and manipulate semantic information. Speech is a serial dynamic auditory signal that needs to be integrated through time, and this is especially true for narratives. Moreover, computing the correct meaning requires the selection from multiple competing representations of speech sounds that may have the same or similar sound. In the same way, the selection from the competing representation of speech sounds needs a constant monitoring on the contextual information (Price, 2012). Notably, speech production and speech comprehension share a considerable amount of cognitive operations (Papathanassiou et al., 2000; Price, 2012), consistently with the broader PFC activity found during RP, including VLPFC. The recruitment of the DLPFC and the FPC, other than the VLPFC, could be explained by the role of DLPFC in the monitoring and manipulation of the information, while the FPC activation could be explained by its role in constructing a global coherence, in particular to get the sense of the story from the encoded information (Mar, 2004). Interestingly, the correlation between the number of recalled elements during the first RP and the O_2_Hb/HHb changes was very close to reach significance, strongly suggesting the presence of a task-related activation. In fact, even though HR changes revealed a significant effect of the time, suggesting a possible effect of the task in the subjects’ anxiety and hemodynamic perfusion, no difference was found in the “state anxiety” before and after the protocol. Moreover, no correlation was found between HR and O_2_Hb/HHb changes (for both EP and RP), showing that the HR and the hemodynamic changes where different in their time courses (Figure 2).

Notably, the PFC activation in response to WM tasks has been widely investigated with fNIRS, both in healthy subjects and in patients (for reviews see Cutini et al., 2012a; Ehlis et al., 2014). For instance, during the execution of a visual *n*-back task utilizing task-relevant and task-irrelevant faces, Schreppel et al. (2008) found that relevant stimuli activated the middle frontal/pre-central cortices and left post-central cortex bilaterally, while irrelevant stimuli activated superior, middle and inferior parts of the right PFC. Such pattern is consistent with the recruitment of a verbal rehearsal strategy to maintain the features of the relevant stimuli and with the selective inhibition needed to properly perform the task, respectively. This is in line also with other neuroimaging studies which demonstrated that PFC activity during WM tasks reflects processes of maintenance, selection and inhibition of information, as well as attentional monitoring (Fletcher and Henson, 2001). On top of that, it has been shown that the hemodynamic response in the PFC and in the temporal regions during an *n*-back WM task may act as a biological marker of social functioning in patients suffering of late onset depression (Pu et al., 2011). WM deficits and PFC dysfunction has also been found in patients with major depressive disorder (Pu et al., 2012). Moreover, Koike et al. (2013) have compared the cortical activation of patients that suffer of schizophrenia with healthy subjects activations during the execution of an *n*-back task, observing in the first a bilateral DLPFC and FPC activation, and in the second a bilateral VLPFC activation accompanied with a task-related deactivation in the DLPFC. Similarly, previous fNIRS studies have shown activation patterns in the VLPFC and DLPFC regions in healthy volunteers performing *n*-back tasks (Ehlis et al., 2008; Pu et al., 2011). To date, the only fNIRS study investigating PFC activity in specific relation to encoding and retrieval processes has been conducted by Okamoto et al. (2011). In particular, they investigated the cortical activity during encoding and retrieval in episodic memory processing of taste information. A cortical activation during taste retrieval was found significantly stronger than that observed during encoding in the bilateral FPC and right DLPFC regions, particularly in the right hemisphere.

The combination of the neuropsychological tests with fNIRS is particularly useful since it enables to monitor the hemodynamic task-related responses while the tests are simultaneously performed. Although the LMT has not been applied yet in the clinical diagnostic field in combination with fNIRS monitoring, it has the potential to be effectively adopted in patients with cognitive disorders and/or WM deficits. For example, Niu et al. (2013) have shown that WM and cognitive abilities, as well as functional deficits in frontal and temporal cortices, can be found in patients with mild cognitive impairment. The importance of identifying these cognitive deficits in the early stages of disease has been widely pointed out (e.g., Lecardeur et al., 2013; Pfeiffer et al., 2013). More specifically, narrative recall tasks, such as the LMT, are included in most standard neurological examinations, and provide a solid approach to elicit semi-structured spontaneous language data, as well as quantity (number of recalled elements) and quality (accuracy and coherence of the retellings) of information. Narrative recall ability is associated with a variety of neurodegenerative and developmental disorders, and it is considered a good predictor of a variety of cognitive and developmental problems as language impairments (Botting, 2002; Dodwell and Bavin, 2008; Duinmeijer et al., 2013), autisms (Tager-Flusberg, 1995; Diehl et al., 2006), dementia (Gomez and White, 2006; Roark et al., 2011), as well as a useful tool to improve the accuracy in the detection of, for example, mild cognitive impairment (Lehr et al., 2012).

Crucially, fNIRS provides neuroscientists with new possibilities for cortical investigations, given its very high experimental flexibility with respect to other neuroimaging methods. Compared with fMRI, fNIRS can simultaneously record the variations of HHb and O_2_Hb concentrations, with a higher temporal resolution, potentially providing a more detailed picture of cortical hemodynamics (e.g., Brigadoi et al., 2012; Cutini et al., 2012b, 2014; Szűcs et al., 2012). Furthermore, fNIRS is silent, more tolerant to subtle movement artifacts (for instance overt speech is allowed), it allows long-time continuous measurements and repeated measurements within short intervals, and offers the possibility to monitor the cortical activity in natural experimental settings.

Nevertheless, the fNIRS technique presents also some limitations that have been previously discussed (Dieler et al., 2012; Quaresima et al., 2012; Scholkmann et al., 2014). The task-evoked changes occurring in forehead skin perfusion could represent an overestimation of the cortical changes, as measured by fNIRS. Recent reports have raised a question against the assumption that PFC O_2_Hb/HHb changes originated only from the cortical hemodynamic response (Kohno et al., 2007; Gagnon et al., 2011, 2012; Takahashi et al., 2011; Kirilina et al., 2012). Such task-evoked changes could result either from systemic blood pressure changes or from skin-specific regulation mechanisms different from the HR autonomic control. In the present study, the subject’s HR time course showed a different pattern in comparison with the time course of O_2_Hb/HHb changes (Figure 2). However, forehead skin perfusion changes would have occurred during the LMT. This potential confounder has been previously investigated by others measuring simultaneously fNIRS signals and forehead skin flow (by a laser Doppler meter) during cognitive tasks (Kohno et al., 2007; Takahashi et al., 2011; Kirilina et al., 2012; Funane et al., 2014). Unfortunately, the costly laser Doppler skin flow meter is not widely available in most of the laboratories, as in the case of the laboratory in which the present study has been carried out. Although several instrumental and/or analysis methods have been proposed to partly account for extracerebral hemodynamic trends in fNIRS signals (Kirilina et al., 2012, 2013; Hallacoglu et al., 2013; Funane et al., 2014), no consensus has been reached yet on the best strategy to be adopted in order to minimize this effect and/or separate superficial and cortical fNIRS responses. Very recently, Kirilina et al. (2013) have proposed a de-noising method that significantly improves the sensitivity of fNIRS to cerebral signals; in that study they combined concurrent time-domain fNIRS and peripheral physiology recordings (mean arterial blood pressure, HR, and skin blood flow) with wavelet coherence analysis. Depth selectivity was achieved by analyzing moments of photon time-of-flight distributions provided by time-domain fNIRS. In the future, the possibility to use time-domain fNIRS systems, combined with peripheral physiology recordings, would offer the advantage to eliminate the extracerebral hemodynamic trends in the fNIRS signals of neurocognitive studies (Torricelli et al., 2014). In terms of data analysis, two or more short-separation channels (as recently suggested by Gagnon et al., 2014) were not included in the layout of the probe holder commercially available for the continuous wave fNIRS instrument utilized in the present study. Therefore, the suppression of the potential superficial artifacts, using for example an additional systemic predictor in the general linear model analysis of the fNIRS data (Gagnon et al., 2011, 2012), was not possible. In addition, it has been reported that the extracerebral contribution is more pronounced in the O_2_Hb than in the HHb signal (Kirilina et al., 2012). In the present study, significant changes in O_2_Hb and HHb were found, and, as suggested by Takahashi et al. (2011), the superficial effect was minimized by an accurate “measurement setting” (see Section Materials and Methods). In particular, the flexible probe holder and its position on the head allowed the creation of a stable optical contact. Although the pressure under the probe was not measured by a membrane pressure sensor, as in the study by Takahashi et al. (2011), the pressure created by the velcro brand fastener was adequate to induce a partial transient blockage of the skin circulation during the present fNIRS study. Although no correction algorithm has been utilized, the higher amplitude of the cortical responses observed over the 16 measurement points during the EP/RP (Figure 2) in comparison with that one observed in the respective control tasks, the unrelated time course of the HR changes (Figure 2), the partial transient blockage of the skin circulation under the fNIRS optodes, and the adequate mean penetration depth of the utilized near-infrared light (source-detector distance was estimated about a half of the 3.5) could support the argument that the described PFC activation during the LMT is mainly a task-related change in PFC oxygenation. On top of that in the present study most of the potential confounders/contamination factors for the fNIRS signals have been consistently reduced by subtracting the PFC oxygenation changes associated with the EPC/RPC from those associated with the EP/RP, respectively. This subtraction procedure has removed also possible inter-individual anatomical differences (i.e., scalp depth) over the investigated PFC areas. Thus, even if a contribution of physiological noise on the broad cortical activity pattern observed during RP cannot be completely ruled out, its influence on hemodynamic activity should be negligible due to the aforementioned subtraction procedures. More importantly, the selective activation found in channels 7 and 15 (Figure 3) during the EP cannot be explained by extracerebral hemodynamic trends. In fact, during the EP the activation was significantly prominent in VLPFC with respect to the other PFC areas, and with respect to the RP. Taken together, we believe that the observed differences in the hemodynamic response over the investigated PFC areas are likely to be specifically bound to the encoding and retrieval processes.

For what concerns the protocol, some methodological considerations should be pointed out on the partial transient blockage of the skin circulation provoked by the 14 optodes. PFC is even involved in pain perception and modulation (Lorenz et al., 2003). Therefore, during fNIRS studies it is important that the probe holder, equipped with the optodes, does not cause any discomfort to the subjects (e.g., physical and/or psychological discomfort). In the present study, in order to avoid any PFC activation induced by discomfort and/or pain, the subjects were properly instructed to alert the researchers whenever they experienced any discomfort and/or pain during the fNIRS measurements. All subjects completed successfully the LMT recording. Moreover, several 60-min fNIRS measurements were carried out separately to evaluate any discomfort and/or pain induced by the probe holder and the optodes. At the end of those fNIRS measurements, no pain was recognized by all the subjects using a numeric rating scale (Turk and Melzack, 2010). Therefore, discomfort and/or pain did not interfere with the LMT-related cortical activation observed in the present study.

In order to suppress any potential interference due to the modalities of the EP and the RP, the EPC and RPC were subtracted to the corresponding tasks. During the EPC the subjects listened the LMT story presented backward; in this case neither memory nor language comprehension were required, and any PFC activity would be related only to the auditory processing of the information. During the RPC the subjects were asked to repeat aloud the week days, repetitively (automatic speech). In this case, the subjects did not have to draw at WM for retrieving the encoded information, but only the vocalization/articulator movements of speech production were required. Taking into account the biphasic time course of O_2_Hb/HHb changes during LMT (Figure 2), the selection of the maximum values of the O_2_Hb/HHb concentration changes during EP and RP was considered the most appropriate for data analysis. We also note some limitations of the present study: the number of participants was limited and the task order was not counterbalanced across subjects, so in future researches we are planning to extend the sample number, also counterbalancing the task order. Moreover, given the restricted PFC area that could be explored using the 16 channel fNIRS system, we note that it might be useful to extend the optical recordings to the whole PFC and to other cortical regions that could be involved in verbal WM processing, such as temporal regions.

In conclusion, this study has demonstrated that, in response to the LMT, PFC oxygenation increases in both hemispheres in healthy subjects. While the EP elicited a markedly selective activity of VLPFC, the activation during the RP was more widespread (including VLPFC, DLPFC, and FPC). This could be explained by the role of the VLPFC in maintaining the information in WM and in understanding the narrative, while the broader PFC activity observed during RP (including DLPFC), could be caused by the need of manipulating and retrieving the memory traces from the previous encoded information in order to perform the task. Thus, the FPC recruitment could be ascribed to the construction of a global coherence essential for the LMT story recall.

To conclude, the results of the present study confirm that fNIRS could be considered as a valuable diagnostic tool, because of its proved applicability for the hemodynamic monitoring during the LMT performance. Considering the necessity of expanding the existing types, quantity and quality of fNIRS paradigms that could induce a cortical activation (Ehlis et al., 2014), future studies should be focused on these purposes, adopting cortical activation tasks as the currently used LMT paradigm.

## Conflict of interest statement

The authors declare that the research was conducted in the absence of any commercial or financial relationships that could be construed as a potential conflict of interest.

## Footnotes

^1^www.mccauslandcenter.sc.edu/mricro/mricron/index.html

## References

[B1] Abikoff H. Alvir J. Hong G. Sukoff R. Orazio J. Solomon S. (1987). Logical memory subtest of the Wechsler memory scale: age and education norms and alternate-form reliability of two scoring systems. J. Clin. Exp. Neuropsychol. 9, 435–448 10.1080/01688638708405063 3597734

[B2] Atkinson R. C. Shiffrin R. M. (1968). “Human memory: a proposed system and its control processes,” in The Psychology of Learning and Motivation, Vol. 2, eds Spence K. W. Spence J. T. (New York: Academic Press), 89–195

[B3] Baddeley A. D. (2012). Working memory: theories, models and controversies. Annu. Rev. Psychol. 63, 1–29 10.1146/annurev-psych-120710-100422 21961947

[B4] Baddeley A. D. Hitch G. (1974). “Working memory,” in The Psychology of Learning and Motivation, ed Bower G. A. (New York: Academic Press), 47–89

[B5] Bayles K. A. (2003). Effects of working memory deficits on the communicative functioning of Alzheimer’s dementia patients. J. Commun. Disord. 36, 209–219 10.1016/s0021-9924(03)00020-0 12742668

[B6] Botting N. (2002). Narrative as a tool for the assessment of linguistic and pragmatic impairments. Child Lang. Teach. Ther. 18, 1–21 10.1191/0265659002ct224oa

[B7] Braun A. R. Guillemin A. Hosey L. Varga M. (2001). The neural organization of discourse: an H2 15O-PET study of narrative production in English and American sign language. Brain 124, 2028–2044 10.1093/brain/124.10.2028 11571220

[B8] Brigadoi S. Cutini S. Scarpa F. Scatturin P. Dell’Acqua R. (2012). Exploring the role of primary and supplementary motor areas in simple motor tasks with fNIRS. Cogn. Process. 13, S97–S101 10.1007/s10339-012-0446-z 22806646

[B9] Buckner R. L. Koutstaal W. (1998). Functional neuroimaging studies of encoding, priming and explicit memory retrieval. Proc. Natl. Acad. Sci. U S A 95, 891–898 10.1073/pnas.95.3.891 9448256 PMC33813

[B10] Cabeza R. Nyberg L. (2000). Imaging cognition II: an empirical review of 275 PET and fMRI studies. J. Cogn. Neurosci. 12, 1–47 10.1162/08989290051137585 10769304

[B11] Cowan N. (2005). Working Memory Capacity. Hove, East Sussex, UK: Psychol Press

[B12] Craik F. I. M. (2002). Levels of processing: past, present… and future? Memory 10, 305–318 10.1080/09658210244000135 12396643

[B13] Crozier S. Sirigu A. Lehéricy S. van de Moortele P. F. Pillon B. Grafman J. (1999). Distinct prefrontal activations in processing sequence at the sentence and script level: an fMRI study. Neuropsychologia 37, 1469–1476 10.1016/s0028-3932(99)00054-8 10617267

[B14] Cutini S. Basso Moro S. Bisconti S. (2012a). Functional near infrared optical imaging neuroscience: an introductory review. J. Near Infrared Spectrosc. 20, 75–92 10.1255/jnirs.969

[B15] Cutini S. Scarpa F. Scatturin P. Dell’Acqua R. Zorzi M. (2012b). Number-space interactions in the human parietal cortex: enlightening the SNARC effect with functional near-infrared spectroscopy. Cereb. Cortex [Epub ahead of print]. 10.1093/cercor/bhs321 23081883

[B16] Cutini S. Scatturin P. Basso Moro S. Zorzi M. (2014). Are the neural correlates of subitizing and estimation dissociable? An fNIRS investigation. Neuroimage 85, 391–399 10.1016/j.neuroimage.2013.08.027 23973407

[B17] Cutini S. Scatturin P. Zorzi M. (2011). A new method based on ICBM152 head surface for probe placement in multichannel fNIRS. Neuroimage 54, 919–927 10.1016/j.neuroimage.2010.09.030 20851195

[B18] Delignières D. (1993). “La perception de l’effort et de la difficulté,” in Cognition Et Performance, ed Famose J. P. (Paris: INSEP Publications), 183–218

[B19] D’Esposito M. Postle B. R. Rypma B. (2000). Prefrontal cortical contributions to working memory: evidence from event-related fMRI studies. Exp. Brain Res. 133, 3–11 10.1007/978-3-642-59794-7_2 10933205

[B20] Diehl J. J. Bennetto L. Young E. C. (2006). Story recall and narrative coherence of high-functioning children with autism spectrum disorders. J. Abnorm. Child Psychol. 34, 87–102 10.1007/s10802-005-9003-x 16485176

[B21] Dieler A. C. Tupak S. V. Fallgatter A. J. (2012). Functional near-infrared spectroscopy for the assessment of speech related tasks. Brain Lang. 121, 90–109 10.1016/j.bandl.2011.03.005 21507475

[B22] Dodwell K. Bavin E. L. (2008). Children with specific language impairment: an investigation of their narratives and memory. Int. J. Lang. Commun. Disord. 43, 201–218 10.1080/13682820701366147 17852521

[B23] Duinmeijer I. de Jong J. Scheper A. (2013). Narrative abilities, memory and attention in children with a specific language impairment. Int. J. Lang. Commun. Disord. 47, 542–555 10.1111/j.1460-6984.2012.00164.x 22938065

[B24] Duncan A. Meek J. H. Clemence M. Elwell C. E. Fallon P. Tyszczuk L. (1996). Measurement of cranial optical path length as a function of age using phase resolved near infrared spectroscopy. Pediatr. Res. 39, 889–894 10.1203/00006450-199605000-00025 8726247

[B25] Ehlis A. C. Bähne C. G. Jacob C. P. Herrmann M. J. Fallgatter A. J. (2008). Reduced lateral prefrontal activation in adult patients with attention-deficit/hyperactivity disorder (ADHD) during a working memory task: a functional near-infrared spectroscopy (fNIRS) study. J. Psychiatr. Res. 42, 1060–1067 10.1016/j.jpsychires.2007.11.011 18226818

[B26] Ehlis A. C. Schneider S. Dresler T. Fallgatter A. J. (2014). Application of functional near-infrared spectroscopy in psychiatry. Neuroimage 85, 478–488 10.1016/j.neuroimage.2013.03.067 23578578

[B27] Ferrari M. Quaresima V. (2012). A brief review on the history of human functional near-infrared spectroscopy (fNIRS) development and fields of application. Neuroimage 63, 921–935 10.1016/j.neuroimage.2012.03.049 22510258

[B28] Fletcher P. C. Henson R. N. (2001). Frontal lobes and human memory: insights from functional neuroimaging. Brain 124, 849–881 10.1093/brain/124.5.849 11335690

[B29] Funane T. Atsumori H. Katura T. Obata A. N. Sato H. Tanikawa Y. (2014). Quantitative evaluation of deep and shallow tissue layers’ contribution to fNIRS signal using multi-distance optodes and independent component analysis. Neuroimage 85, 150–165 10.1016/j.neuroimage.2013.02.026 23439443

[B30] Fuster J. M. (2002). “Physiology of executive function: the perception-action cycle,” in Principles of Frontal Lobe Function, eds Stuss D. T. Knight R. T. (New York: Oxford University Press), 96–108

[B31] Gagnon L. Cooper R. J. Yücel M. A. Perdue K. L. Greve D. N. Boas D. A. (2012). Short separation channel location impacts the performance of short channel regression in NIRS. Neuroimage 59, 2518–2528 10.1016/j.neuroimage.2011.08.095 21945793 PMC3254723

[B32] Gagnon L. Perdue K. Greve D. N. Goldenholz D. Kaskhedikar G. Boas D. A. (2011). Improved recovery of the hemodynamic response in diffuse optical imaging using short optode separations and statespace modeling. Neuroimage 56, 1362–1371 10.1016/j.neuroimage.2011.03.001 21385616 PMC3085546

[B33] Gagnon L. Yücel M. A. Boas D. A. Cooper R. J. (2014). Further improvement in reducing superficial contamination in NIRS using double short separation measurements. Neuroimage 85, 127–135 10.1016/j.neuroimage.2013.01.073 23403181 PMC3665655

[B34] Gazzaley A. Nobre A. C. (2012). Top-down modulation: bridging selective attention and working memory. Trends Cogn. Sci. 16, 129–135 10.1016/j.tics.2011.11.014 22209601 PMC3510782

[B35] Gomez R. G. White D. A. (2006). Using verbal fluency to detect very mild dementia of the Alzheimer type. Arch. Clin. Neuropsychol. 21, 771–775 10.1016/j.acn.2006.06.012 17011743

[B36] Graesser A. C. Hauft-Smith K. Cohen A. D. Pyles L. D. (1980). Advanced outlines, familiarity and text genre on retention of prose. J. Exp. Edu. 48, 281–290

[B37] Hallacoglu B. Sassaroli A. Fantini S. (2013). Optical characterization of two-layered turbid media for non-invasive, absolute oximetry in cerebral and extracerebral tissue. PLoS One 8:e64095 10.1371/journal.pone.0064095 23724023 PMC3660388

[B38] Kane M. J. Bleckley M. K. Conway A. R. Engle R. W. (2001). A controlled-attention view of working-memory capacity. J. Exp. Psychol. Gen. 130, 169–183 10.1037/0096-3445.130.2.169 11409097

[B39] Kelley W. M. Miezin F. M. McDermott K. B. Buckner R. L. Raichle M. E. Cohen N. J. (1998). Hemispheric specialization in human dorsal frontal cortex and medial temporal lobe for verbal and 28 nonverbal memory encoding. Neuron 20, 927–936 10.1016/S0896-6273(00)80474-280474-2 9620697

[B40] Kirilina E. Jelzow A. Heine A. Niessing M. Wabnitz H. Brühl R. (2012). The physiological origin of task-evoked systemic artefacts in functional near infrared spectroscopy. Neuroimage 61, 70–81 10.1016/j.neuroimage.2012.02.074 22426347 PMC3348501

[B41] Kirilina E. Yu N. Jelzow A. Wabnitz H. Jacobs A. M. Tachtsidis I. (2013). Identifying and quantifying main components of physiological noise in functional near infrared spectroscopy on prefrontal cortex. Front. Hum. Neurosci. 7:864 10.3389/fnhum.2013.00864 PMC3865602 24399947

[B42] Kohno S. Miyai I. Seiyama A. Oda I. Ishikawa A. Tsuneishi S. (2007). Removal of the skin blood flow artifact in functional near-infrared spectroscopic imaging data through independent component analysis. J. Biomed. Opt. 12, 062111 10.1117/1.2814249 18163814

[B43] Koike S. Takizawa R. Nishimura Y. Kinou M. Kawasaki S. Kasai K. (2013). Reduced but broader prefrontal activity in patients with schizophrenia during n-back working memory tasks: a multi-channel near-infrared spectroscopy study. J. Psychiatr. Res. 47, 1240–1246 10.1016/j.jpsychires.2013.05.009 23743135

[B44] Lecardeur L. Meunier-Cussac S. Dollfus S. (2013). Cognitive deficits in first episode psychosis patients and people at risk for psychosis: from diagnosis to treatment. Encephale 39, S64–S71 10.1016/j.encep.2012.10.011 23528322

[B45] Lehr M. Prud’hommeaux E. Shafran I. Roark B. (2012). Fully automated neuropsychological assessment for detecting mild cognitive impairment. Proceedings of the 13^th^ Annual Conference of the International Speech Communication Association.

[B46] Lorenz J. Minoshima S. Casey K. L. (2003). Keeping pain out of mind: the role of the dorsolateral prefrontal cortex in pain modulation. Brain 126, 1079–1091 10.1093/brain/awg102 12690048

[B47] Mar R. A. (2004). The neuropsychology of narrative: story comprehension, story production and their interrelation. Neuropsychologia 42, 1414–1434 10.1016/j.neuropsychologia.2003.12.016 15193948

[B48] Mazziotta J. Toga A. Evans A. Fox P. Lancaster J. Zilles K. (2001). A probabilistic atlas and reference system for the human brain: international consortium for brain mapping (ICBM). Philos. Trans. R. Soc. Lond. B. Biol. Sci. 356, 1293–1322 10.1098/rstb.2001.0915 11545704 PMC1088516

[B49] Niu H. J. Li X. Chen Y. J. Ma C. Zhang J. Y. Zhang Z. J. (2013). Reduced frontal activation during a working memory task in mild cognitive impairment: a non-invasive near-infrared spectroscopy study. CNS Neurosci. Ther. 19, 125–131 10.1111/cns.12046 23279823 PMC6493442

[B50] Oberauer K. (2009). Design for a working memory. Psychol. Learn. Motiv. 51, 45–100 10.1016/S0079-7421(09)51002-X

[B51] Okamoto M. Wada Y. Yamaguchi Y. Kyutoku Y. Clowney L. Singh A. K. (2011). Process-specific prefrontal contributions to episodic encoding and retrieval of tastes: a functional NIRS study. Neuroimage 54, 1578–1588 10.1016/j.neuroimage.2010.08.016 20832483

[B52] Oldfield R. (1971). The assessment and analysis of handedness: the Edinburgh inventory. Neuropsychologia 9, 97–113 10.1016/0028-3932(71)90067-4 5146491

[B53] Otten L. J. Rugg M. D. (2001). Task-dependency of the neural correlates of episodic encoding as measured by fMRI. Cereb. Cortex 11, 1150–1160 10.1093/cercor/11.12.1150 11709486

[B54] Papathanassiou D. Etard O. Mellet E. Zago L. Mazoyer B. Tzourio-Mazoyer N. (2000). A common language network for comprehension and production: a contribution to the definition of language epicenters with PET. Neuroimage 11, 347–357 10.1006/nimg.2000.0546 10725191

[B55] Pearce P. Johnson C. Manly P. Locke J. (2013). Use of narratives to assess language disorders in an inpatient pediatric psychiatric population. Clin. Child Psychol. Psychiatry [Epub ahead of print]. 10.1177/1359104513487001 23689481

[B56] Petrides M. (1989). “Frontal lobe and memory,” in Handbook of Neuropsychology, eds Boller F. Grafman J. (Amsterdam, New York: Elsevier), 601–614

[B57] Pfeiffer H. C. Løkkegaard A. Zoetmulder M. Friberg L. Werdelin L. (2013). Cognitive impairment in early-stage non-demented Parkinson’s disease patients. Acta Neurol. Scand. [Epub ahead of print]. 10.1111/ane.12189 24117192

[B58] Postle B. R. (2006). Working memory as an emergent property of the mind and brain. Neuroscience 139, 23–38 10.1016/j.neuroscience.2005.06.005 16324795 PMC1428794

[B59] Price C. J. (2012). A review and synthesis of the first 20 years of PET and fMRI studies of heard speech, spoken language and reading. Neuroimage 62, 816–847 10.1016/j.neuroimage.2012.04.062 22584224 PMC3398395

[B60] Pu S. Yamada T. Yokoyama K. Matsumura H. Kobayashi H. Sasaki N. (2011). A multi-channel near-infrared spectroscopy study of prefrontal cortex activation during working memory task in major depressive disorder. Neurosci. Res. 70, 91–97 10.1016/j.neures.2011.01.001 21241745

[B61] Pu S. Yamada T. Yokoyama K. Matsumura H. Mitani H. Adachi A. (2012). Reduced prefrontal cortex activation during the working memory task associated with poor social functioning in late-onset depression: multi-channel near-infrared spectroscopy study. Psychiatry Res. 203, 222–228 10.1016/j.pscychresns.2012.01.007 22964135

[B62] Quaresima V. Bisconti S. Ferrari M. (2012). A brief review on the use of functional near-infrared spectroscopy (fNIRS) for language imaging studies in human newborns and adults. Brain Lang. 121, 79–89 10.1016/j.bandl.2011.03.009 21507474

[B63] Roark B. Mitchell M. Hosom J. P. Hollingshead K. Kaye J. (2011). Spoken language derived measures for detecting mild cognitive impairment. IEEE Trans. Audio Speech Lang. Processing 19, 2081–2090 10.1109/tasl.2011.2112351 22199464 PMC3244269

[B64] Robertson D. A. Gernsbacher M. A. Guidotti S. J. Robertson R. R. Irwin W. Mock B. J. (2000). Functional neuroanatomy of the cognitive process of mapping during discourse comprehension. Psychol. Sci. 11, 255–260 10.1111/1467-9280.00251 11273413 PMC4301434

[B65] Rugg M. D. Johnson J. D. Park H. Uncapher M. R. (2008). Encoding–retrieval overlap in human episodic memory: a functional neuroimaging perspective. Prog. Brain Res. 169, 339–352 10.1016/S0079-6123(07)00021-0 18394485

[B66] Scholkmann F. Kleiser S. Metz A. J. Zimmermann R. Mata Pavia J. Wolf U. (2014). A review on continuous wave functional near-infrared spectroscopy and imaging instrumentation and methodology. Neuroimage 85, 6–27 10.1016/j.neuroimage.2013.05.004 23684868

[B67] Schreppel T. Egetemeir J. Schecklmann M. Plichta M. M. Pauli P. Ellgring H. (2008). Activation on the prefrontal cortex in working memory and interference resolution processes assessed with near-infrared spectroscopy. Neuropsychobiology 57, 188–193 10.1159/000147473 18654088

[B68] Shankle W. R. Romney A. K. Hara J. Fortier D. Dick M. B. Chen J. M. (2005). Methods to improve the detection of mild cognitive impairment. Proc. Natl. Acad. Sci. U S A 102, 4919–4924 10.1073/pnas.0501157102 15781874 PMC555034

[B69] Spielberger C. D. Gorsuch R. L. Lushene R. Vagg P. R. Jacobs G. A. (1983). Manual for the State-Trait Anxiety Inventory. Palo Alto, CA: Psychologists Press

[B70] Szűcs D. Killikelly C. Cutini S. (2012). Event-related near-infrared spectroscopy detects conflict in the motor cortex in a Stroop task. Brain Res. 1477, 27–36 10.1016/j.brainres.2012.08.023 22921848

[B71] Tager-Flusberg H. (1995). Once upon a ribbit: stories narrate by autistic children. Br. J. Dev. Psychol. 13, 45–59 10.1111/j.2044-835x.1995.tb00663.x

[B72] Takahashi T. Takikawa Y. Kawagoe R. Shibuya S. Iwano T. Kitazawa S. (2011). Influence of skin blood flow on near-infrared spectroscopy signals measured on the forehead during a verbal fluency task. Neuroimage 57, 991–1002 10.1016/j.neuroimage.2011.05.012 21600294

[B73] Torricelli A. Contini D. Pifferi A. Caffini M. Re R. Zucchelli L. (2014). Time domain functional NIRS imaging for human brain mapping. Neuroimage 85, 28–50 10.1016/j.neuroimage.2013.05.106 23747285

[B74] Turk D. C. Melzack R. (2010). The Handbook of Pain Assessment - Third Edition. New York: The Guildford Press

[B75] Uncapher M. R. Otten L. J. Rugg M. D. (2006). Episodic encoding is more than the sum of its parts: an fMRI investigation of multifeatural contextual encoding. Neuron 52, 547–556 10.1016/j.neuron.2006.08.011 17088219 PMC1687210

[B76] Wechsler D. (1997). Wechsler Memory Scale – Third Edition Manual. San Antonio, TX: The Psychological Corporation

[B77] Wechsler D. (2008). Wechsler Adult Intelligence Scale. San Antonio, TX: The Psychological Corporation

